# Assessing the generalisation of artificial intelligence across mammography manufacturers

**DOI:** 10.1371/journal.pdig.0000973

**Published:** 2025-08-12

**Authors:** Alistair J. Hickman, Sandra Gomes, Lucy M. Warren, Nadia A.S. Smith, Caroline Shenton-Taylor

**Affiliations:** 1 Department of Physics, University of Surrey, Guildford, United Kingdom; 2 Department of Scientific Computing and National Co-ordinating Centre for the Physics of Mammography, Royal Surrey NHS Foundation Trust, Guildford, United Kingdom; 3 National Physical Laboratory, Teddington, United Kingdom; University of Pennsylvania, UNITED STATES OF AMERICA

## Abstract

The aim of this study was to determine whether differences between manufacturer of mammogram images effects performance of artificial intelligence tools for classifying breast density. Processed mammograms from 10,156 women were used to train and validate three deep learning algorithms using three retrospective datasets: Hologic, General Electric, Mixed (equal numbers of Hologic, General Electric and Siemens images) and tested on four independent witheld test sets (Hologic, General Electric, Mixed and Siemens). The area under the receiver operating characteristic curve (AUC) was compared. Women aged 47-73 with normal breasts (routine recall - no cancer) and Volpara ground truth were selected from the OPTIMAM Mammography Image Database for the years 2012-2015. 95 % confidence intervals are used for significance testing in the results with a Bayesian Signed Rank test used to rank the overall performance of the models. Best single test performance is seen when a model is trained and tested on images from a single manufacturer (Hologic train/test: 0.98 and General Electric train/test: 0.97), however the same models performed significantly worse on any other manufacturer images (General Electric AUCs: 0.68 & 0.63; Hologic AUCs: 0.56 & 0.90). The model trained on the mixed dataset exhibited the best overall performance. Better performance occurs when training and test sets contain the same manufacturer distributions and better generalisation occurs when more manufacturers are included in training. Models in clinical use should be trained on data representing the different vendors of mammogram machines used across screening programs. This is clinically relevant as models will be impacted by changes and upgrades to mammogram machines in screening centres.

## Author summary

A number of manufacturers of mammogram machines are in use within the NHS Breast Screening Program. Naturally some of these manufacturers use different technologies to acquire the mammograms. These mammograms are made readable through the application of processing to the raw information from the X-ray detector, which is known to vary both inter- and intra- manufacturer. The aim of this study was to assess whether these differences impact the performance of AI classification algorithms. We trained three binary classifiers on three different datasets, two from single manufacturers and one with an even mix of three manufacturers. Models trained on single manufacturer data could not generalise their knowledge to manufacturers unseen in training. The model trained on three manufacturers was the best overall performer. In general models must be trained on images from any manufacturers in the desired clinical setting as there are sufficient differences between manufacturers that AI algorithms cannot transfer their knowledge to a mammogram from an unseen manufacturer. Models must also be monitored and kept up to date to reflect any changes to mammogram machines within the clinical setting.

## Introduction

AI systems have been shown to be effective when performing tasks on mammograms but these studies are often single institution or single manufacturer [1–6]. These studies assess AI models on both processed and unprocessed mammograms. Within the NHS Breast Screening Program (NHSBSP) mammogram systems from multiple vendors are used, these different systems use a range of detector types and different vendors apply different processing algorithms to the unprocessed images so they can be read by radiologists creating a range of vendor - processing algorithm combinations. Evidence has already shown that difference image processing impacts the rates of microcalcification detection by radiologists in digital mammograms [7]. As such performance differences of AI tools on processed images from different manufacturers is to be anticipated. Due to storage costs unprocessed images are not routinely stored within the NHSBSP. The knowledge of whether deep learning algorithms can generalise across processed images from different mammogram machine vendors is an important prerequisite to clinical deployment.

It is well known that women with high breast density have a higher risk of masking with mammography imaging and a higher risk of breast cancer [8]. Therefore, it would be useful to be able to identify the women with the highest breast density for supplemental screening with other imaging techniques. Commonly used methods of breast density estimation include estimation by radiologists and calculation with commercial software. Estimation by radiologists suffers from large inter- and intra- observer variability [9,10]. One piece of commercial software (Densitas Inc, Halifax, NS, Canada) and a few research softwares [2,3,11,12] have been developed to calculate breast density on processed images.

An AI binary classification system has been developed to make breast density predictions from processed mammograms with the positive class being defined as the 75^*th*^ percentile. This study aims to determine whether differences between manufacturers affect the classification performance of a deep learning system. This research is part of a wider question as to whether generalised models are possible. A study by de Vries [13] found software versions for a single model of mammography equipment affected recall rates and that ‘per-software version’ thresholds are required. This knowledge is a core part of clinical deployment across healthcare systems in which equipment vendors can differ between screening centres. The evaluation of performance across manufacturers is the novel aspect of this work.

## Materials and methods

### Data collection

In the NHSBSP in the UK women are invited for screening every three years between the ages of 50 and 70 years. An on-going randomised controlled trial is investigating the use of age extension to 47-73 years [14]. During the screening examination, two views of both breasts are acquired using a mammography system, Craniocaudal and Mediolateral Oblique.

The data used in this study is from on the OPTIMAM Mammography Image Database [15]. Images for this database are collected from eight screening sites which are used for the NHSBSP. Images are stored in standard DICOM format and identifying information is anonymised in accordance with DICOM Supplement 142 Annex AI. Images were selected from the database for women with normal breasts (routine recall – no cancer) who were screened between 1st January 2014-31st December 2014. In addition, a random selection of 25% of all women (with routine recall - no cancer) screened in 2012, 2013, 2015 were collected. These dates were chosen to provide a sample across a whole single year and representative samples across three other years. Some DICOM tags are Pseudonymised to allow annotation of images by radiologists. These processes have received ethical approval [REC Reference 19/SC/0284] . Data were collected with approval from an ethical research committee specialising in research databases organised by the NHS Health Research Authority. All images used in this study were de-identified at the point of collection, and therefore written informed consent from the patients was not required.

### Ground truth

The ground truth breast density was calculated for all images using Volpara software version 1.5.4 (VolparaSolutions Limited, Wellington 6011, NZ), outputting the volumetric breast density. The 75^*th*^ percentile in volumetric breast density was identified as 9.37 % over the entire study population. The women were classified as either above or below this threshold. These two classes are referred to as ‘Dense’ and ‘Non-Dense’ respectively. The 75^*th*^ percentile was chosen rather than the mean density. Holland [16] reported that interval cancers were disproportionately found in women with higher breast densities. Due to the scale of the NHSBSP, the top quartile has been chosen as it covers the highest risk women classified as having ‘extremely dense’ and there are no studies assessing cost effectiveness of additional screening. The boundary selected is lower than the 38.5 % discussed by Holland due to the scale of the NHSBSP, whilst any artificial intelligence methods would need be automated as to not add unnecessary burdens, both financial and human resource.

### Study data selection

**Fig 1:**
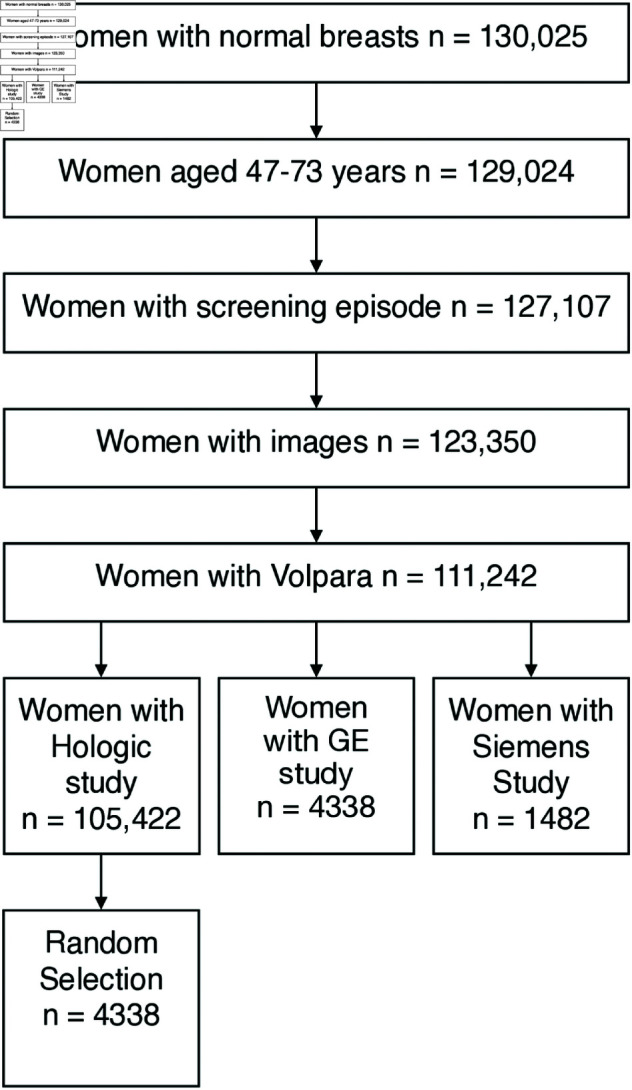
Data selection flowchart. Images were collected to the database for all women with normal breasts (routine recall – no cancer) screened between 1st January 2014 and 31st December 2014. In addition, a random selection of 25 % of all women screened in 2012, 2013 & 2015 were collected. A random selection of those with Hologic studies and Volpara ground truths were chosen for the study.

Women with ‘Normal’ breasts (no cancer), aged 47-73 years, with Volpara ground truth were selected for the study (as shown in Fig 1). The women were split by manufacturer of X-ray imaging equipment – Siemens (Siemens AG, Berlin, Germany), GE (General Electric Company, Boston, USA) and Hologic (Hologic Inc., Malborough, USA).

### Pre-processing and model architecture

Information on pre-processing and the model architecture can be found in S1 Appendix.

The parameters of the model were optimised using the training and validation sets. The best performance was assessed in terms of the area under the ROC curve and accuracy in the two classes and the recall of the positive class on the validation data sets. The trained models were then tested on witheld test sets.

### Performance evaluation

A deep learning algorithm was trained and validated on three datasets. One containing only GE images (referred to as ‘GE’), one containing only Hologic images (‘Hologic’) and one containing an even mixture of Hologic, GE and Siemens images (‘Mixed’). The three models were then tested on four withheld test datasets; one Hologic only, one GE only, one Siemens only (‘Siemens’) and one containing an even mixture of Hologic, GE and Siemens images. There is no overlap of images between any two datasets. There is insufficient data to produce Siemens only train and validation data sets. The total number of women contained within the datasets can be seen in Table 1. No woman appears more than once in the study, there is no overlap between any datasets and all images from the selected screening study (both views of both breasts) are used within the study and are provided as separate inputs to the models.

**Table 1:** The distribution of women across the Training, Validation and Test datasets. The named manufacturer datasets consist of images solely from that manufacturer whilst the Mixed datasets are made up of even distributions of Hologic, GE and Siemens images.

	Hologic	GE	Siemens	Mixed
Train	2169	2169	-	2169
Validation	542	542	-	540
Test	540	540	395	552

It is important for our study to estimate the variance in performance of classifiers trained on different datasets from the same manufacturer. The variance in performance due to intra-manufacturer data selection can be compared to the variance due to inter-manufacturer data to show the former is insignificant. For this purpose, we also investigated the variance in performance of our algorithm as a result of being trained on five independent Hologic datasets. These were made up of 54,377 images from 13,554 women, none of whom were included in any other data set.

### Statistical analysis

A Chi-Squared test is used to test for statistically significant differences between the demographic means. Bootstrapping was used to generate 1000 samples with replacement which were used to generate 95 % confidence intervals for the AUC values. Statistical significance decision were made using overlaps in the 95 % CI values.

Advice on the best method to compare overall classifier performance differs across researchers in the literature [17–20]. As Demsar [21] noted, all of the most popular techniques to generate performance estimates will generate samples that violate the independent samples assumption of most statistical tests, and hence lead to statistical designs which are only approximate.

Due to these limitations in the overall performance is assessed using a Bayesian Signed Rank test as discussed by Benavoli [20]. This is done using the ‘Baycomp’ software presented in the same study. This test does not require synthetic samples and is able to provide meaningful results with the real samples within the study.

## Results

Table 2 contains the demographics of women in the study, separated by manufacturer. The women with Siemens images were significantly older(p = 0.0001), had breasts which were significantly thinner and had a higher volumetric breast density (p = 0.0001) than women with Hologic or GE images (p = 0.0001).

**Table 2:** Table showing the average age, compressed breast thickness and volumetric breast density of the patients. The volumetric breast density has been assessed by Volpara Solutions. The value provided is the mean with the standard deviation in brackets. The overall average breast density top quartile value was used across the study.

	Hologic	GE	Siemens
Number of Women	4338	4338	1482
Age (years)	59.7 (0.02)	59.4 (0.2)	62.0 (0.02)
Compressed Breast Thickness (mm)	56.5 (0.02)	58.4 (0.12)	52.4 (0.15)
Volumetric Breast Density (%)	7.72 (0.05)	7.23 (0.04)	8.86 (0.08)
Thresholds for breast density quartiles (%)			
Q1/Q2	4.26	3.65	4.44
Q2/Q3	5.96	5.56	6.87
Q3/Q4	9.37	9.05	11.5

### Models trained and tested on datasets with the same manufacturer distributions

**Fig 2:**
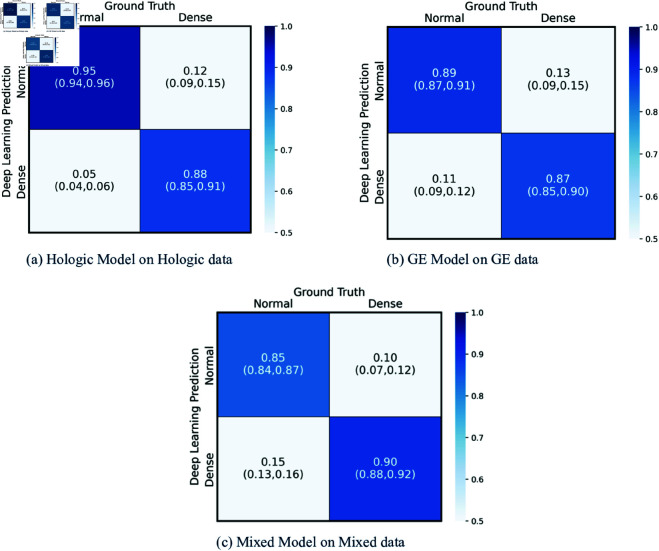
Confusion matrices models tested on same data distribution as training. Confusion matrices showing the performance of the three models when trained, validated and tested on the same respective manufacturer. The performance is shown using True Negatives and False Negatives along the top row and False Positives and True Positives along the bottom row of each heat map with 95 % confidence intervals shown in brackets.

The confusion matrices for the three trained models when tested on the independent test datasets with the same distribution of manufacturers as their training datasets are shown in Fig 2. The rate of correct ‘Dense’ classifications can be seen in the lower right quadrant. The models predicted these with 88 %, 87 % and 90 % for Hologic, GE and Mixed data respectively. The values in brackets are the 95 % confidence intervals, these were calculated using 1000 bootstrap samples.

### Models trained and tested on a range of manufacturers

**Fig 3:**
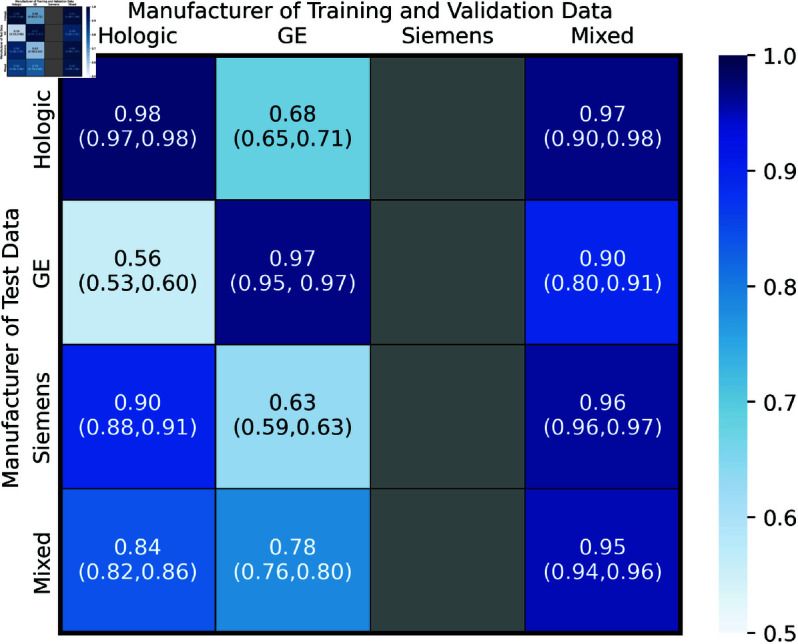
AUC Heatmap of all train/test permutations. Heatmaps showing the AUC values of each of the three models on the corresponding 4 witheld test sets. The 95 % Confidence Intervals are shown in brackets and were calculated from 1000 bootstrap samples.

The Hologic, GE and Mixed models were tested on the four independent test datasets; Hologic only, GE only, Siemens only and an equal mix of all three. The areas under the ROC curves are shown in Fig 3.

#### Performance of the same model across different datasets.

Both the Hologic and GE models performed significantly better when tested on their corresponding Hologic only and GE only datasets compared with any of the other three test datasets. The model trained and validated on Mixed data performed significantly worse on the GE only test set compared to the Siemens only and Mixed test sets. The difference in performance of the Mixed model between the GE only test set and Hologic only test set was not significant. The error on these two results are large compared to the errors of all the other results.

#### Comparing different models across the same dataset.

When testing on the Hologic only test data the Hologic and Mixed model performed similarly, but both performed significantly better than the GE model. When testing on GE only data the GE model performed significantly better than the Mixed model and both performed significantly better than the Hologic model. When testing on Siemens images the Mixed model performed significantly better than the Hologic model which performed significantly better than the GE model. When testing on the Mixed data set the Mixed model performed significantly better than the Hologic model which performs significantly better than the GE model.

Performance on test data from unseen manufacturers (Hologic on GE and Siemens only, GE on Hologic and Siemens only) was only good in one of the four cases, Hologic Model on Siemens only data. This suggests GE images present differently to the AI compared to Hologic and Siemens images which are more similar.

These results show there are sufficient differences between images from different mammography vendors as to impact the performance of a deep learning model. It is unknown whether this results from the processing algorithms used or the difference in detectors used to acquire the images. All the models developed are affected by differences in vendor. Optimal single dataset performance in seen for the Hologic and GE models tested on the Hologic only and GE only test datasets respectively. The most generalisable model is the Mixed model but even this sees reduced performance on GE only images. This suggests tailored models per manufacturer is ideal. Some generalisation is seen and the reduction in performance of the Mixed on the GE only images may be mitigated by increasing the share of GE images in training.

### Overall performance and statistical analysis

Table 3 shows the probability that a given model exhibits overall better performance across all test datasets than another. There is a 75.2 % chance that the Mixed model is overall better than the Hologic model, a 96.1 % chance that the Mixed model is better than the GE model and there is a 74.9 % chance that the Hologic model is better than the GE model. These results imply that the Mixed model is the most generalisable, followed by Hologic and finally the GE model is the least. This suggests that including a greater number of manufacturers in training improves the generalisation across manufacturers.

**Table 3:** Results of Bayesian Signed Rank test. The rows are pairwise comparisons of the models and the outputs are given that the probability that the first is better, equal or worse than the second model. Mixed (Mix), Hologic (Hol), General Electric (GE).

	P(better)	P(equal)	p(worse)
Mix vs Hol	0.752	0.006	0.241
Mix vs GE	0.961	0.004	0.035
Hol vs GE	0.749	0.007	0.244

### Variance

In order to assess the variance in performance five further Hologic models were trained on 5 training and validation datasets and tested on the same Hologic only test set. Due to the abundance of Hologic data these datasets are independent from both each other and the original training and validation datasets. The same algorithm was used to create these models. These produced AUC values of 0.978, 0.979, 0.974, 0.981 and 0.982 with a mean of 0.979 and standard deviation of 0.003. The variance observed in training models is much smaller than the difference between values observed from a single model tested on different test datasets. This shows the results observed are due to differences within the processed images rather than due to any variance in the algorithm’s training.

### Overall performance and statistical analysis

Table 4 shows the probability that a given model exhibits overall better performance across all test datasets than another. There is a 75.2 % chance that the Mixed model is overall better than the Hologic model, a 96.1 % chance that the Mixed model is better than the GE model and there is a 74.9 % chance that the Hologic model is better than the GE model. These results imply that the Mixed model is the most generalisable, followed by Hologic and finally the GE model is the least. This suggests that including a greater number of manufacturers in training improves the generalisation across manufacturers.

**Table 4:** Results of Bayesian Signed Rank test. The rows are pairwise comparisons of the models and the outputs are given that the probability that the first is better, equal or worse than the second model. Mixed (Mix), Hologic (Hol), General Electric (GE).

	P(better)	P(equal)	p(worse)
Mix vs Hol	0.752	0.006	0.241
Mix vs GE	0.961	0.004	0.035
Hol vs GE	0.749	0.007	0.244

## Discussion

The models trained on images from a single manufacturer perform significantly better when tested on the same single manufacturer data compared to images from other manufacturers. Comparatively the model trained on images from three mainstream manufacturers performed worse on the GE only images compared to any of the other three test datasets. The mixed model exhibited the greatest generalisation and this may be further improved by using more GE data. To our understanding this is the first study to compare the performance of a deep learning algorithm to predict breast density across different manufacturers. A study by Van Vugt *et al.* [12] trained a computer aided detection algorithm on Hologic images and measured the performance on Siemens images using transfer images. The study does not compare to performance with the same manufacturer images and suffers from small numbers of cases. Zanca *et al.* [7] performed a similar study assessing detection of microcalcifications by radiologists. They found ‘image processing has a significant impact on the detection of microcalcifications in digital mammograms’ by radiologists although the magnitude of this effect is smaller with radiologists than with AI.

The main limitation of this study is a result of data limitations. The limited availability of GE and Siemens studies meant the sizes of the data sets for the GE only and Mixed models were limited, thus limiting the size of the Hologic only training set, despite an abundance of data. There was also not enough Siemens data for a Siemens only training set for additional comparisons and the Siemens only test set was smaller than the others. The requirement that the Mixed dataset contains even numbers of images from the three manufacturers limits its size and thus the size of the Hologic and GE only datasets are subject to the same limit. The study would benefit from a greater number of manufacturers but data was not available from others in sufficient quantity and those included in the study represent the largest in the NHSBSP. The data included is from years 2012-2015, this does not cover recent changes in equipment and any future studies should endeavour to include more recent images.

The exact reason for the difference in performance is unknown. Alongside the different processing algorithms, manufacturers use different detectors. These produce images of different image size and pixel size, 70μm for the Amorphous Selenium detector used by Hologic and Siemens and 100μm for the Caesium Iodide phosphor used by GE [22]. The contribution of each difference is unknown and is a possible avenue for future work. This could be explored through a similar study that used unprocessed (‘For Processing’) mammograms. Alternative methods of overcoming the problem such as domain adaptation should be explored.

A significant challenge, particularly within healthcare is the task of updating AI models upon release of new hardware and software. In Healthcare settings time is required to build up data in order to update models. This is an ongoing research question and guidance is required on avoiding this problem.

## Conclusion

They key clinical finding from this study is that deep learning models trained on images from a single manufacturer performed significantly worse on datasets containing images from other manufacturers. This implies that if a model is to be used in a clinical setting it should be trained on data from all manufacturers used within that setting to maximise its performance. There can be no certainty that a model can generalise between processed images from any two manufacturers nor that it can generalise between different software versions. Care must be taken that a model is able to perform well in all locations and conditions in which it is being deployed and that models reflect any changes or upgrades to the X-ray systems within even individual screening centres.

## Supporting information

S1 Appendix Image pre-processing and model design.
(PDF)

S1 Fig CNN Model Architecture. The number of features written are the number output after the application of 0.5 dropout.
(TIFF)
