# Supplementary material for: Assessing the generalisation of artificial intelligence across mammography manufacturers
Source: PLOS Digit Health. 2025 Aug 12;4(8):e0000973. doi: 10.1371/journal.pdig.0000973 (PMC12342238; doi:10.1371/journal.pdig.0000973)
Supplement: S1 Appendix — Image pre-processing and model design. (PDF) [file pdig.0000973.s001.pdf]

S1 Appendix

Processed ('For Presentation') mammograms were further pre-processed to be used as input data. Images were resized to the same aspect ratio. The images were padded to the largest image width and image height (5253 x 4041 pixels) to maintain the same aspect ratio as the most common image size, to avoid distorting the images. This maintained the relative area of the breast. The images were then resized to the most common size (3328 x 2560 pixels) to reduce computation expenditure.

The mean and standard deviation of the training images for each manufacturer were calculated. This was used during training and validation to normalise the images to zero mean and unit standard deviation as is common in these types of studies [1]. No other augmentation or pre-processing was performed.

The classes are inherently imbalanced due to the selection of the top quartile so a weighted Cross Entropy Loss function was used [2]. Better performance on the validation set was observed with weights of ratio 1:5 (non-dense:dense), a ratio greater than the 1:3 conventional wisdom would suggest.

earning rates were calculated using a tool based off work by Smith [3], these were calculated to be 2e-5.The weights were initialised by randomly drawing them from a gaussian distribution with standard deviation of sqrt(2/n).

Model Design

The structure of the five layer CNN used can be seen in S1 Fig. This model was created using PyTorch(version 2.0.0). The dimensions of each layer refer to the [filter size x the stride] used for the filter. The activation functions for each layer are not shown. The first two convolutional layers use a ReLU (Rectified Linear Unit) activation function whilst the final three use ELU (Exponential Linear Unit) activation functions.

S1 Fig. CNN Model Architecture. The number of features written are the number output after the application of 0.5 dropout.

The first layer is an average pooling layer to downsample the image using a 4x4 kernal, then a convolution layer only, followed by three convolution layers with max pooling. Finally, a fully connected layer provided a confidence score for the predictions of the two classes 'Dense' and 'Non-Dense'. ReLU and ELU activations were used alongside an Adam optimiser and a dropout of 0.5.

References

1. LeCun Y, Bottou L, Orr G, Muller KR. Neural Networks: Tricks of the Trade. 2nd ed. Berlin: Springer; 2012.

2. Rezaei-Dastjerdehei M , Mijani A, Fatemizadeh E. Addressing Imbalance in Multi-Label Classification Using Weighted Cross Entropy Loss Function. 2020 27th National and 5th International Iranian Conference on Biomedical Engineering (ICBME).

3. Smith L. No More Pesky Learning Rate Guessing Games. CoRR
