# Supplementary figures and images for: Assessing the generalisation of artificial intelligence across mammography manufacturers
Source: PLOS Digit Health. 2025 Aug 12;4(8):e0000973. doi: 10.1371/journal.pdig.0000973 (PMC12342238; doi:10.1371/journal.pdig.0000973)

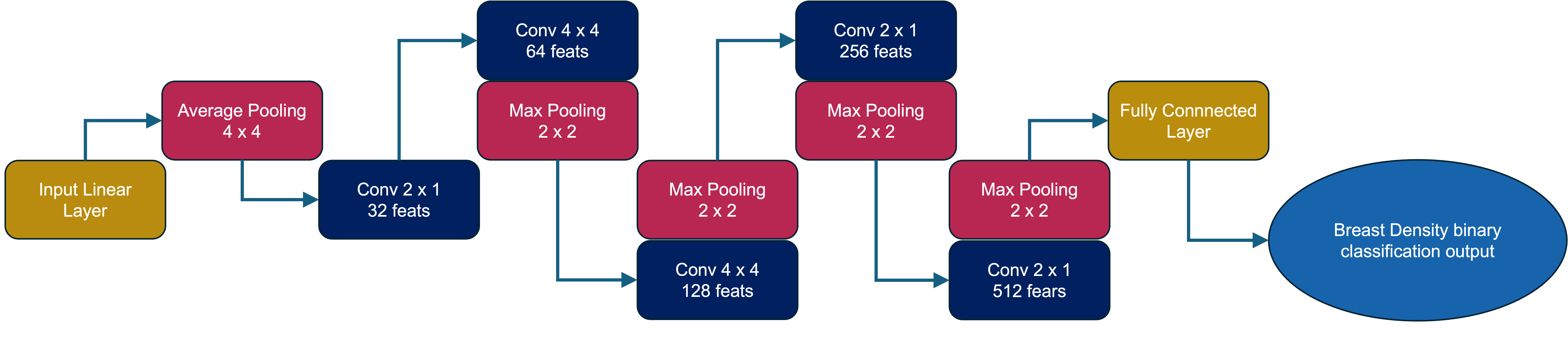

Supplement: S1 Fig — CNN Model Architecture. The number of features written are the number output after the application of 0.5 dropout. (TIFF) [file pdig.0000973.s002.tiff]
